# Role of Exosomes in Cardiovascular Diseases

**DOI:** 10.31083/j.rcm2506222

**Published:** 2024-06-19

**Authors:** Qiumei Lin, Pingfeng He, Jing Tao, Jing Peng

**Affiliations:** ^1^Department of General Medicine, Chongqing University Central Hospital, Chongqing Emergency Medical Center, Chongqing Key Laboratory of Emergency Medicine, 400014 Chongqing, China

**Keywords:** exosomes, biomarkers, therapeutic, cardiovascular diseases, intracellular communication

## Abstract

Exosomes (EXOs) are a subgroup of extracellular vesicles (EVs) that contain numerous biologically active molecules. They exhibit an essential mode of cell communication, primarily between distinct cell populations, for the maintenance of tissue homeostasis and coordination of adaptive responses to various stresses. These intercellular communications are vital for the complex, multicellular cardiovascular system. In the last ten years, their potential role as effective tissue-to-tissue communicators has received increasing attention in cardiovascular physiology and pathology. There is growing evidence that repair of the heart and regeneration can be promoted by EXOs derived from cardiomyocytes or stem/progenitor cells. However, the underlying mechanisms remain unclear. EVs derived from different stem/progenitor cell populations have been used as cell-free therapies in different preclinical models involving cardiovascular diseases and have shown promising results. In this review, we have summarized the recent developments in EXOs research, the impact of EXOs derived from different cells on the cardiovascular system, their potential therapeutic roles as well as new diagnostic biomarkers, and the possible clinical translational outcomes.

## 1. Introduction

The cardiovascular system is composed of different cell types that form a dynamic network that functions in supporting blood circulation throughout the body [1]. The heartbeat is induced by electric signals coupled with contractile movements, which propel blood into the vessels which are essential in regulating perfusion and fluid balance. The continuous adaptation of these characteristics to environmental changes necessitates a multifaceted and tight system of intercellular communication and transcriptome modulation [2]. In multicellular organisms, in addition to intercellular communication via tight, physical, intercellular connections, paracrine signaling allows intercellular communication through the unidirectional or bidirectional transfer of signaling molecules [3, 4]. Extracellular vesicles (EVs), encompassing a broad categorization of cell-derived membranous structures, are encapsulated within lipid bilayers and are inherently non-replicative, and devoid of functional nuclei. Currently, this field lacks a unified consensus regarding specific biomarkers for the differential categorization of EV subpopulations. Over the past decade, the role of EVs as vital mediators of intercellular communication, and their involvement in the transmission of biological signals between prokaryotic and higher eukaryotic cells to regulate a variety of biological processes, have been identified [5].

All EV subtypes consist of a specific molecule confined within a surrounding lipid bilayer of varied sizes and buoyant densities [6]. Confusion in the nomenclature of vesicles exists due to the variety of vesicles released from the cells and the numerous approaches utilized to isolate them. EVs are composed of exosomes, microvesicles, and apoptotic bodies. Exosomes are distinguished by a diameter of 30–100 nm, whereas microvesicles have a diameter of 100–1000 nm. In contrast, apoptotic bodies are characterized by a diameter of 500–4000 nm. These EVs vary in their biological source, markers, secretion methods, and contents [7]. Exosomes are EVs that have particular patterns of messenger RNA (mRNA), micro RNA (miRNA), long non-coding RNAs, and in some instances, genomic DNA. Their function in cellular transportation is based on the transfer of genetic information, which in turn triggers persistent or transient phenotypic changes in the recipient cell [8, 9]. Exosomes possess pleiotropic biological properties such as antigen presentation, immune response, intracellular communication, and RNA and protein transfer. Currently, there is abundant evidence that exosomes can mediate paracrine, autocrine, and endocrine functions [10]. Exosomes of various myocardial cells take part in the processes of angiogenesis, apoptosis, cell migration, proliferation, hypertrophy, and regeneration. An expanding body of research highlights significant distinctions between exosomes secreted by cells in hypoxic versus normoxic conditions. Hypoxia not only augments the secretion of EVs, but also modulates their molecular cargo, including alterations in RNA, protein, and other biomolecular constituents within the EVs. This mechanistic adaptation allows hypoxia to influence the functional dynamics and behavioral responses of recipient cells [11, 12, 13, 14]. Recently, the potential role of EVs as effective inter-tissue communicators in the physiology and pathology of the cardiovascular system has received increasing attention [15]. One area for the application of exosomes for clinical usage is diagnostic: exosomes derived from the blood or urine can be utilized as disease and prognostic markers for cancer, and potentially for central nervous system diseases and heart disease. In addition, exosomes derived from distinct stem/progenitor cells have been found to have therapeutic value [16]. We explored the current literature on exosome biogenesis, structure, content, and their impact from multiple cell types on the cardiovascular system, their potential diagnostic role in cardiovascular diseases, and their role as a novel therapeutic weapon for cardiovascular diseases. We have also reviewed the engineering of endogenous exosomes and presented some novel methods for the generation and design of synthetic exosomes that deliver therapeutic agents to the heart.

## 2. An Overview of Exosomes

EVs are produced in response to different stimuli including cell differentiation, activation, aging, lack of oxygen, and viral infection [17]. EVs secreted by cells include microvesicles, exosomes, and apoptotic bodies [18]. Exosomes are a subgroup of EVs with a diameter of 30 to 100 nm. They are released by distinct cell types. These exosomes, based on their origin and size, can be differentiated from both apoptotic and microvesicle bodies (Table 1) [19]. Exosomes are bilayer membrane vesicles that were discovered in sheep serum in 1979 and named “exosome” by Johnstone in 1987. Intracellular lysosomal microparticles form exosomes and these exosomes can be found in mammalian blood, urine, and ascites, where they can be used for diagnostic as well as prognostic evaluations [20, 21]. The only known secretory cell vesicles that originate from the inner membrane are the exosomes. They are essentially endoplast multivesicular bodies (MVBs) inward budding intralumenal vesicles (ILVs) targeting the plasma membrane [22]. The prevailing view at the time was that these vesicles facilitated the removal of plasmalemma factors that were no longer required by mature red blood cells [23]. However, with the evolving understanding of paracrine mechanisms, these particles that were once considered to be implicated in waste management are now extensively accepted as elements involved in short- and long-range communication pathways, playing an important role in intercellular communication [2]. Exosomes are produced by the activity of several factors in the endosomal system, and several signaling molecules are isolated into the exosomal cavity during these processes [24]. Exosomes originate from the inward budding of the plasma membrane of early endosomes [25]. Initially, the plasma membrane undergoes early inward budding within the body, embedded in particular membrane proteins. Consequently, further inward budding of the endosomal membrane produces several ILVs. These early endosomes are called MVBs [26]. Upon maturation, MVBs may either merge with lysosomes for degradation or integrate with the plasma membrane, facilitating the release of ILVs, commonly referred to as exosomes. The genesis of exosomes is intricately linked to the selective incorporation of cargo molecules, a process controlled by diverse intracellular complexes through two primary routes: the endosomal sorting complexes required for transport (ESCRT)-dependent pathway and the ESCRT-independent pathway. The ESCRT-dependent mechanism unfolds sequentially, engaging various ESCRT subcomplexes, including Tumor Susceptibility Gene 101 (TSG101) and Committee for Medicinal Products (CHMP) proteins. Conversely, the ESCRT-independent pathway encompasses the ceramide-driven process, leading to the formation of membrane subdomains and the aggregation of tetraspanin proteins such as cluster of differentiation (CD)63, CD81, and CD9. These aggregates promote inward vesicular budding and the genesis of EVs. Exosomes acquire their vesicular constituents via either the ESCRT-dependent or independent mechanisms. The ESCRT machinery, a constellation of cytoplasmic proteins, is instrumental in the biogenesis of MVBs, facilitating membrane invagination and the sorting of proteins (both ubiquitinated and non-ubiquitinated), lipids, and nucleic acids into ILVs [27]. Exosomes consist of a lipid bilayer enveloping a small organelle-free cytoplasm that contains a heterogeneous arrangement of large molecules [5]. Transmission electron microscopic studies reveal that the exosomes are either round/spherical or cup-shaped [28]. They possess various types of RNA molecules, such as circular RNA (circRNA), mRNA, long non-coding RNA (lncRNA), and miRNA [29].

**Table 1.** **Characteristic parameters of different size-based EV subtypes**.

	Exosomes	Microvesicles	Apoptotic bodies
Size	30 to 100 nm	100 to 1000 nm	500 to 4000 nm
Density	1.13–1.19 g/mL	Unknown	1.16–1.28 g/mL
EM morphology	Cup shaped	Heterogeneous	Heterogeneous
Cellular Origin	Most cell types	Most cell types	All cell types
Origin	Plasma membrane endosomes	Plasma membrane	Plasma membrane endoplasmic reticulum
Composition	Biochemical composition known, but most proteins and lipids not unique for exosomes	Insufficiently known	Histones, DNA

EV, extracellular vesicle; EM, electron microscopy.

**Fig. 1.**
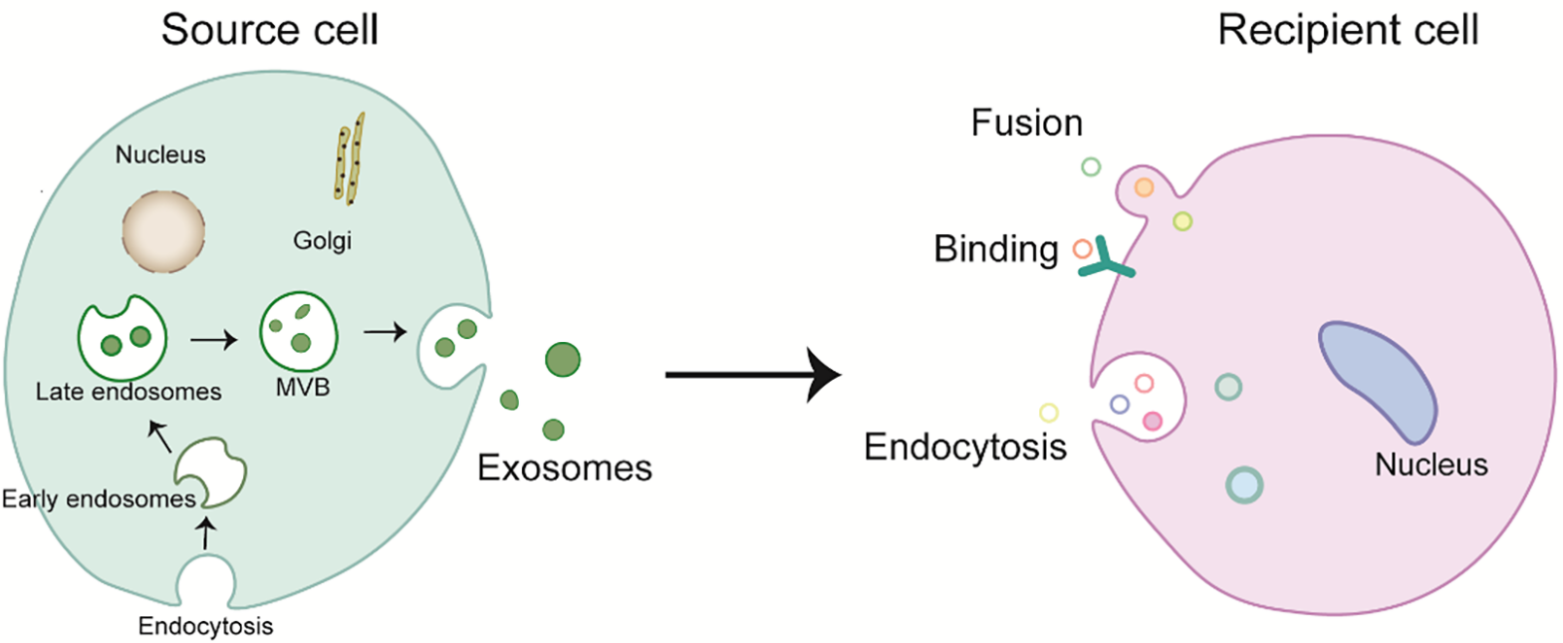
**Biogenesis and internalization mechanism of exosomes. **MVB, multivesicular bodies.

EVs, secreted across a spectrum of cellular entities, play pivotal roles in physiological processes, involving immune modulation and cellular discourse. They serve as a fundamental mechanism for the transfer of intercellular information. Studies have demonstrated that EVs contribute to both the physiological and pathological paradigms of organismal function, with the potential for utilization in the diagnosis and prognostic assessment of diseases. They reach the recipient cell and affect its gene expression and, in some cases, its function [30]. Binding, fusion, and endocytosis are the three types of internalization mechanisms that describe the uptake of exosomes by recipient cells (Fig. 1) [31]. The binding of exosomes to recipient cells makes them function externally as ligands (i.e., without incorporation into the cell) and activate receptor-mediated signal transduction. When they fuse directly to the recipient cell membrane, their cellular content is released into the cytoplasm of the recipient cell [32]. This movement of cellular content from one cell to the other helps regulate many biological events, such as antigen presentation, coagulation, proliferation, differentiation, immune cell signaling, angiogenesis, wound healing, regeneration, growth and organ development, and pathological changes [33]. The bioactive molecules contained within the exosomes are thought to (1) directly trigger the target cells through the bioactive lipids present in the exosome or via soluble cell surface signaling complexes; (2) transfer the carcinogenic substances as well as cancer cell properties of the exosome to nearby inert or normal cells; (3) play a role in the epigenetic reprogramming of the recipient cells by transferring mRNA, miRNA, and transcription factors [34]. The transport of exosomes between various cells can affect the physiological pathways of the recipient cells. Their composition varies according to the source of the cell type. A recent study on exosomes suggests that they may contain multiple elements in addition to proteins, such as various nucleic acids including DNA, mRNA, miRNA, and lncRNA [35].

Research on the involvement of EVs, especially exosomes, under different physiological and pathological conditions, has gained interest over the last decade [36]. Exosomes were originally thought to be molecular messengers, functioning through autocrine and paracrine signaling and causing a phenotypic change in the receptor cell [37]. Several recent studies have shown that these vesicles play a significant role in various biological functions. Exosomes have been implicated in antigen presentation [38], neuronal communication [39, 40], wound healing, blood coagulation [41], mature sperm [42], and the modulation of immunological responses against the fetus during pregnancy under physiological conditions. They have also been implicated in cancer [43], autoimmune diseases [44], infection, inflammation [45], and metabolic and cardiovascular diseases (CVD) [46].

## 3. Exosomes Secreted by Different Cell Types

Various cells constitute the heart including cardiomyocytes, endocardial cells, fibroblasts, epicardial cells, inflammatory cells, and immune cells [47]. The intensive communication of these cells is through direct cell-to-cell contact and via paracrine interactions to enhance normal heart function (Table 2) [48]. Studies have demonstrated that exosomes are synthesized by the majority of heart cells, blood vessel cells, and myocardial stem cells [49, 50, 51, 52, 53]. In myocardial tissue, cardiac fibroblasts represent the predominant cell type within the non-muscle cellular compartment, comprising approximately 90% of these cells. Upon interaction with these cells, endothelial cells (ECs) play a key role in cardiac homeostasis. Studies have found the existence of resident cardiogenic progenitor cells in post-injury responses [54]. Their presence in the heart affirms the significance of heterocellular communication and the need to further study the mechanism underlying this communication [48, 55]. In cardiovascular diseases, exosomes derived from cardiac cells influence the exchange of molecular signals and carriers to activate the target molecules, which regulate inflammatory factors and ultimately promote cardiac regeneration and function [56]. Therefore, exosomes are prime candidates for the mediation of communication between various cell populations [57]. Exosomes derived from non-cardiovascular cells can also affect the physiological and pathological states and function of the cardiovascular system.

**Table 2.** **Exosomes secreted by various cells and their roles**.

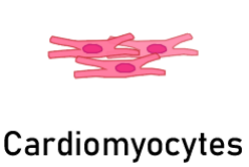	lncRNA AK139128	Promote apoptosis and inhibit cell proliferation in CFs *in vitro* and *in vivo*
	miR-208a	Promote fibroblast proliferation and myofibroblast differentiation
	miR-210-3p	Promote cell proliferation and collagen synthesis by inhibiting GPD1L in atrial fibroblasts
	miRNA-92a	Trigger phenotypic transformation of fibroblasts into myofibroblasts
	miR-320	Inhibit the proliferation, migration and angiogenesis of ECs
	circHIPK3, circHIPK1	Reduce oxidative stress-induced damage and protect cardiac microvascular endothelial cells
	lncRNA-AK139128	Promote apoptosis and inhibit proliferation and migration of myocardial fibroblasts
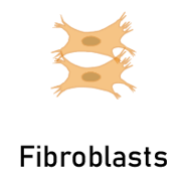	miR-21_3p (miR-21*)	Helps the development of cardiomyocyte hypertrophy
	miR-23a-3p	Promotes iron death and ultimately leads to the occurrence of atrial fibrillation
	miR-200a-3q	Reduce vasogenesis capacity, migration capacity and increase vascular permeability of ECs
	miRNA-423-3p	Reduce heart damage caused by ischemia-reperfusion injury and improve heart function
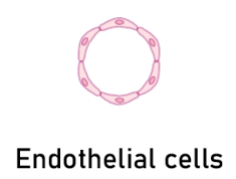	circHIPK3	Promote VSMC proliferation and inhibit VSMC apoptosis
	miR-505	Through the induction of neutrophil extracellular traps formed to aggravate atherosclerosis
	miRNA-143/145	Inhibit the proliferation and migration of SMCs and maintain the integrity of vascular endothelium
	lncRNA-LINC00174	Reduce myocardial injury induced by ischemia-reperfusion
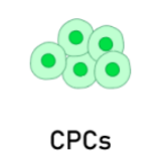	miR-210	Inhibit cardiomyocyte apoptosis
	miR-132	Enhancing tube formation in endothelial cells
	miR-210	Inhibit cardiomyocyte apoptosis, promote angiogenesis and improve cardiac function
	miR-132	Mediate SVP capacity to alleviate interstitial fibrosis in infarcted hearts
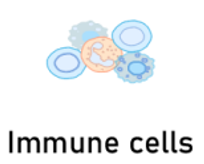	miR-3-4p	Promotes ventricular remodeling after ischemia
	miR-155	Promote the development of atherosclerosis
	lncRNA 39868	Alleviate myocardial oxidative stress injury induced by ischemia-reperfusion
	circRNAbe2a	Aggravate myocardial fibrosis after acute myocardial infarction

CFs, cardiac fibroblasts; ECs, endothelial cells; VSMC, vascular smooth muscle cells; SVP, saphenous vein-derived progenitor cells; CPCs, cardiac progenitor cells; GPD1L, Glycerol-3-Phosphate Dehydrogenase 1 Like; SMCs, smooth-muscle-cell; *, in miR-21 indicates that this miRNA molecule is produced by processing the antisense strand of the miRNA precursor.

## 4. Exosomes Derived from Various Cell Types

### 4.1 Cardiomyocytes

Although cardiomyocytes are not primarily secretory cells, they can release exosomes [58]. Gupta and Knowlton [59] found that exosomes are released by cardiomyocytes, and that these exosomes are released not only during normal physiological conditions but also following hypoxia. The release of exosomes and the amount of heat shock protein (HSP)60 increased during stress conditions [59]. Further studies have shown that the HSP60 in exosomes binds to the membrane and is not released, thus preventing the pro-apoptotic effects induced by the circulating HSP60 [60]. Another study showed that hypoxia induces the upregulation and enrichment of miR-30a in exosomes and that miR-30a is efficiently transferred between hypoxic cardiomyocytes via the exosomes. The inhibition of exosome release was found to be conducive to the maintenance of autophagy after hypoxia [61]. Another study demonstrated the protective effect of exosomes derived from plasma containing HSP70, an exosome marker, against cardiomyocytes (CMs), in ischemia/reperfusion (I/R) models. Exosome HSP70 promotes the survival of CMs by participating in the toll-like receptor 4 (TLR4) signaling pathway [62]. A study by Malik *et al*. [60] suggests that oxygen (reactive oxygen species) and alcohol may increase the exosome secretion capacity of CMs and alter the proteome profile of cardiac EVs. There is growing evidence that EVs released by myocardial cells are transferred by transcribing different DNA sequences into multiple mRNAs [60]. CMs-derived exosomes/microvesicles (MV) are rich in mitochondrial, ribosome, and cytoplasmic transcripts, suggesting that these vesicles are involved in the transport of transcripts involved in energy production [63].

Waldenström *et al*. [64] found that exosomes derived from cardiomyocyte DNA in recipient fibroblasts take part in varied cell-related processes in the recipient fibroblasts by regulating gene expression. Recently, the interaction between CM and cardiac fibroblasts (CF) has been shown to be crucial in the progression of chronic heart failure (CHF) and myocardial fibrosis, contributing to the development of cardiac hypertrophy and decreased myocardial function [65]. The close anatomical and functional connection between CM and EC indicates that CM communicates with EC and vice versa, especially following pathological stress. Exosomes obtained from cardiomyocytes influence the function of cardiomyocytes and therefore affect many of the heart’s physiological and pathological functions [66]. Yuan *et al*. [67] showed that exosomes from cardiomyocytes initiate the repair of the heart following myocardial infarction by transferring multiple functional molecules to their target cells. Additionally, the exosomes convey pro-angiogenic as well as anti-angiogenic factors and thus, are involved in regulating angiogenesis [68]. Collectively, these studies imply that the exosomes produced by cardiomyocytes can convey distinct biomolecules to other cell types and thus, regulate their gene expression.

### 4.2 Fibroblasts

Cardiac fibroblasts comprise approximately 60 to 70% of all normal cardiomyocytes and approximately one-third of the normal cardiac volume [69]. The fibroblast is the predominant cell engaged in extracellular matrix (ECM) regeneration. Because of its secretory activity, it affects the physiology of the other cardiomyocytes [70]. A study reported that normoxia and hypoxia conditions alter the content and number of the CF exosome proteins. Additionally, they also found that hypoxic conditions led to the overexpression of proteins connected to the mitochondria, and hypothesized that cells can use exosomes under stress conditions to remove dysfunctional mitochondria [70]. In addition to proteins, CF exosomes also have miRNAs [71]. Lyu *et al*. [72] studied the role of CF-derived exosomes in the communication between CF and CM and showed that angiotensin II (Ang II) treatment stimulates the production of exosomes from CF and that CM absorbs these exosomes. These exosomes, in turn, upregulate the expression of Ang II and its receptor in CM, thereby enhancing Ang II-associated cardiac hypertrophy [72]. They also showed that the expression of renin, AT1R, Agt, and AT2R is upregulated while that of ACE2 is downregulated by the CF-derived exosomes in cultured neonatal cardiomyocytes. Additionally, they also found that the CF exosomes also stimulate protein kinase B (PKB/Akt), p38 mitogen-activated protein kinase (p38, MAPK), extracellular regulated protein kinases (ERK), and c-Jun N-terminal kinase (JNK) to enhance the synthesis and release of Ang II, which is linked to epidermal growth factor receptor (EGFR) and spp1 [72]. Another study found that CF exosomes have abundant miR-21* (*, miR byproduct) and that the presence of miR-21* in exosomes causes cardiomyocyte hypertrophy [71]. miR-21* overexpression can decrease the expression of SORBS2 (sorbin and SH3 domain 2) and PDLIM5 (PDZ and LIM domain 5) in myocardial cells, whereas the suppression of SORBS2 and PDLIM5 can induce hypertrophy in cardiomyocytes [71]. Therefore, the miR-21* in exosomes can migrate into cardiac cells and result in cardiomyocyte hypertrophy [71]. Exosomes of CF and CM were shown to have increased levels of miR-27a, miR-28-3p, and miR-34a in a congestive heart failure mouse model, all of which inhibit nuclearfactor erythroidderived 2-like 2 (Nrf2) translation [73]. During myocardial injury, the Nrf2/angiotensin converting enzyme (ACE) signaling pathway functions as an antioxidant, delaying the process of cardiac failure and ventricular remodeling [74]. In rat models of myocardial infarction (MI), in contrast to the control group, the application of CF-derived exosomes resulted in a 25% reduction in myocardial injury. Moreover, the presence of CF co-cultured with CM increased the activity of CM after hypoxia and reoxygenation injury in a paracrine-dependent manner [75].

In summary, the exosomes secreted by cardiac fibroblasts can protect against cardiac hypertrophy and the process of fibrosis and exert a protective effect on the heart.

### 4.3 Endothelial Cells

ECs create an endothelial barrier between the blood and surrounding tissues that functions to maintain homeostasis, and after stress signals, during hypoxia and inflammation [76]. In addition to specific exosome markers that express CD63 and CD81, EC-derived exosomes have a variety of EC-specific surface markers, including CD31, CD54, CD62E, CD105, CD144, CD146, and von Willebrand factors [77, 78]. Exosomes are utilized by ECs to communicate with themselves, specifically in the management of angiogenesis. One study found that the delta-like 4 factor (DL-4), a key factor regulating angiogenesis, is present in the exosomes produced by ECs and that these exosomes are absorbed by neighboring ECs [79]. These exosomes promote angiogenesis through EC transfer by inhibiting Notch signaling without the need for intercellular contact [80]. Another study has shown that endothelium-derived particles promote cell survival, counteract the clotting process, exert an anti-inflammatory effect, and induce endothelial regeneration [77]. Exposure to lipopolysaccharide circulating endothelial cells (CEC)-exobiology tends to express integrin αvβ6, resulting in T cells by αvβ6-transforming growth factor-beta (TGF-β) proliferation. Other studies have validated the involvement of TGF-β in the formation and growth of atherosclerotic plaques. A clinical study demonstrated that serum HSP70 levels were inversely associated with CVD, particularly atherosclerosis, hypertension, and coronary vascular disease [81]. It is important to note that, in the past, studies on CEC-derived exosomes emphasized vascular injury and angiogenesis. Nevertheless, CEC-exobiology, CM-exosomes, and CF-exosomes have different functions in CVD.

### 4.4 Cardiac Progenitor Cells

Cardiac progenitor cells (CPCs) were first identified in rat hearts. In the myocardium and cardiac vasculature, CPCs exhibit the capacity to differentiate into major cardiac cells [82]. Progenitor cells lie between adult cells and stem cells. CPCs are considered to be among the most promising stem cells for cardiac regeneration and repair [83]. The cardioprotective effect of CPCs is mainly achieved through paracrine mechanisms to reduce tissue damage and/or promote tissue repair. Exosomes demonstrate a significant function in the paracrine effects of CPCs [84]. A recent study has shown that CPCs have cardioprotective properties as a result of the presence of pregnancy-associated plasma protein-A on their surface, the active form of which cleaves insulin-like growth factor-binding protein-4 (IGFBP-4) and promotes the production of insulin-like growth factor-1 (IGF-1). IGF-1 is a key protective factor of the heart [85]. In 2018, Nie *et al*. [86] published a study on the proteomic and RNA sequencing analyses of the CPC secretome, confirming the pro-angiogenic, pro-survival, and pro-mitotic impacts of these CPC exosomes. They observed increased levels of miRNA precursors and miRNAs that induce cell survival, proliferation, and angiogenesis. In addition, they also found small, long non-coding RNAs [86]. In another study, Ibrahim and colleagues [87] revealed that the exosomes extracted from CDCs possess cardio-protective properties.

### 4.5 Immune Cells

In comparison to cardiomyocytes and stem/progenitor cells, the immune cells are essential for inflammation and immune responses during various CVDs [88]. Both intracellular proteins and extracellular stimuli control the biogenesis, secretion, and uptake of immune cell-derived exosomes [89]. The discovery of the function of immune cells secreted exosomes complicates the study of the involvement of various immune cells in the progression of cardiac disease [90]. Due to the lack of research in this area, it is challenging to ascertain the likely applications of these exosomes in heart repair. However, significant evidence has demonstrated the cardioprotective effect of immune cell-derived exosomes. Exosomes derived from specialized antigen-presenting dendritic cells (DCs) and macrophage have been linked to myocardial infarction, atherosclerosis, cardiomyopathies, and endothelial dysfunction related to hypertension [91, 92]. These specific antigen-presenting vesicles have a remarkably controlled outcome compared to direct cell contact techniques, which may lead to unpredictable side effects [93]. Regulatory T cells (Tregs) are adept at modulating the immune response through exosomal interactions across various immune cell subsets [94]. The complex roles of immune cells in influencing cardiovascular disease outcomes have only recently become a focal point of extensive research. Advancing our knowledge of exosomal mechanisms will inevitably propel the discovery of innovative approaches to cardiac protection [95].

## 5. Diagnostic Potential of Exosomes in Cardiovascular Diseases

To facilitate timely therapeutic interventions, early and accurate diagnosis of CVDs is essential. Exosomes are considered key mediators of health and disease and may be biomarkers for specific diseases [96]. In acute myocardial infarction (AMI), many markers lack specificity because an elevation in their levels can be detected during cardiotoxicity following chemotherapy, pulmonary embolism, chronic renal failure, and non-cardiac surgery [97]. Therefore, additional biomarkers are needed to help rapidly diagnose AMI with high specificity. Exosomes are regarded as excellent diagnostic biomarkers because of their capacity to change their delivery in response to various cell stimuli [98]. They can be utilized in cancer and other diseases to monitor disease progression and assess the response of various treatments. The biogenesis of exosomes is intrinsically linked to the cellular processes of disease. Its bilayer membrane forms through a raft of microdomains rich in sphingolipids, cholesterol, and ceramides, providing remarkable preservation and protection of cargo substances for instance, RNA in body fluids. These characteristics, apart from being easily analyzed and accessible, allow them to be attractive biomarkers for clinical disease diagnosis and prognosis, as demonstrated by the series of studies on cardiovascular diseases [99]. Many miRs are specifically enriched in cardiomyocytes, and these miRs participate in the regulation of cardiac development and function. The levels of these miRs in the plasma of patients with AMI are significantly potentiated. However, these myocardial miRs cannot predict future cardiovascular events or detect the presence of CHF [100]. This investigation found that the serum concentration of miR-192 exhibits a significant elevation in patients with acute AMI who subsequently develop ischemic heart failure (HF). Concurrently, miR-194 and miR-34a levels were also elevated in tandem with miR-192, particularly within exosomes, indicating their collective role as circulating mediators in the pathogenesis of HF through the p53 signaling pathway. Moreover, this study established a significant correlation between the expression levels of miR-194 and miR-34a and left ventricular end-diastolic dimension (LVEDD) one year post-AMI, underscoring their prognostic relevance in cardiac remodeling processes. Another study found that the p53-reactive miRNA levels in the circulating EVs were elevated in patients with AMI in the “subacute phase” (approximately 18 days after myocardial infarction). A different study reported that the levels of miR-423-5p, 22, 320a, and 92b in the serum exosomes were elevated in systolic heart failure patients and that these miRs are important clinical prognostic parameters [101]. Additionally, the proteins in the exosomes can also be utilized as biomarkers to evaluate cardiovascular risk and pathology. The EC marker CD31 is an independent predictor of cardiovascular events in patients with stable coronary artery disease (CAD) [100]. In a double-blind, randomized, sham-controlled study, EVs were recovered from 30 patients who were randomly assigned (1:1) to undergo the RIPC- (EV-RIPC) or sham procedures (EV-naive) before PCI. Patient-derived EVs were analyzed by transmission electron microscope (TEM), fluorescence activated cell sorting (FACS) and western blot, which found that troponin (TnT) was enriched in EVs, compared to healthy subjects, regardless of the diagnosis. This analysis of ACS-patients’ EVs demonstrated that the ability of the *in vitro* assay to predict the effectiveness of EV *in vivo * [102].

## 6. Exosomes as Therapeutic Agents for Cardiovascular Diseases

Exosome transplantation is an effective therapeutic option for cardiovascular diseases. Studies have confirmed that compared with cell transplantation, secreted body transplantation can reduce immune rejection and improve the survival rate [99]. miRNAs, as well as proteins released by different cardiomyocyte exosomes, regulate target gene expression and cellular function. They additionally function by alleviating cardiac hypertrophy, malfunction, and fibrosis while enhancing post-infarction myocardial repair, angiogenesis, and anti-atherosclerotic progression [103]. The possibility of using exosomes as an alternative to whole-cell therapy has received much attention. Exosomes have several advantages over whole cells that are used for therapeutic purposes; they are non-immunogenic, biocompatible, and non-tumorigenic. In addition, they exhibit physiological stability in comparison to whole cells. They can circulate throughout the body, and they possess the ability to pass through the blood-brain barrier (BBB) [104]. Furthermore, they are also suitable for carrying therapeutic material and are highly resistant to freeze-thaw procedures than whole cells, thereby making them suitable for long-term storage [105].

To investigate the cardioprotective as well as heart repair impacts of exosomes, scientists have isolated the exosomes from stem cells from various sources [106]. As mentioned previously, exosomes from CPCs have a regenerative potential, which aids in cardiac regeneration after myocardial infarction. Several studies have shown that they can suppress cell apoptosis in CMs and the development of fibrosis while potentiating tube formation, resulting in improved cardiac function [107]. The expression levels of the CM and EC genes in CPC increase when stimulated by embryonic stem cell (ES)-derived exosomes. The injection of pre-stimulated CPCs increases the function of the heart and minimizes the size of the infarct, while the direct injection of embyonic stem cell microvesicles (ESMVs)/exosomes increases blood vessel density and improves the myocardial function [108]. These studies suggest that the alteration of the donor cell conditions is an effective way to alter exosome content, enhancing its capacity for cardiovascular protection. In addition, pretreatment of stem cells utilizing exosomes can enhance their therapeutic effects. Research is underway to discover other approaches to load additional material into stem cell exosomes to expand their therapeutic potential beyond their natural role. These approaches include endogenous and passive or active exogenous cargo encapsulation. These methods disrupt the lipid bilayer of the exosomes over time, allowing drugs to enter [109].

**Fig. 2.**
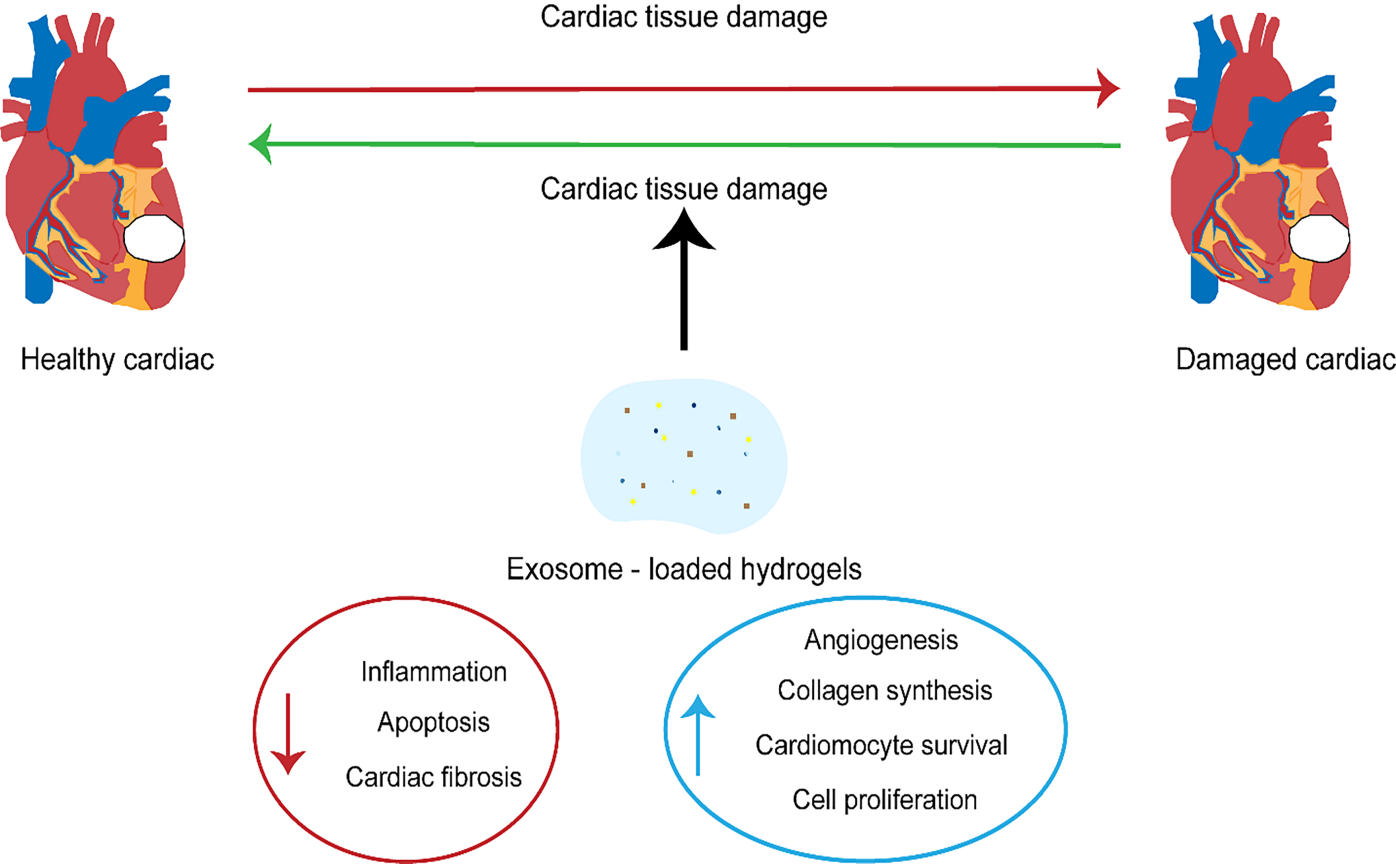
**The therapeutic significance of exosomes in cardiovascular diseases**.

Although there are many advantages to the potential utilization of exosomes in the treatment of cardiovascular diseases, a decisive factor in achieving this goal is finding the right method to deliver the exosomes to make them effective [110]. In cardiovascular therapy, the ideal mode of delivery of exosomes is intravenous; however, studies have shown that this approach results in the absorption of the exosomes by the liver. A comparison of intra-myocardial (IM) and intra-coronary (IC) exosome injections showed that intramuscular injections exhibited higher efficacy than IC injections [111]. One of the biggest drawbacks linked to drug delivery is high retention in the liver, and exosomes targeting the heart are not exempt from this challenge. Nevertheless, engineered exosomes appear to significantly increase the number of cells reaching the target cell in the heart [112] (Fig. 2).

## 7. Conclusions

The field of evaluating exosomes has increased greatly owing to their great potential for disease diagnosis and treatment and in particular, because of their role as effective mediators of inter-tissue crosstalk. The EV biogenesis pathway is unique and closely associated with the network of intracellular transport vesicles involved in most cellular activities in health and disease states. Therefore, exosomes are independent biological vesicles functioning through paracrine mechanisms, contributing to the pathology, repair, and protection of ischemic heart disease. They are also potentially better biomarkers that can convey benefits in the diagnosis and prognostic prediction of diseases. Moreover, since they offer easy accessibility, storage, and can be easily modified, providing exciting avenues for drug delivery and cell-free therapies. Furthermore, preclinical studies using stem/progenitor cell EVs to treat ischemic heart disease have demonstrated increased advantages and fewer adverse events.

However, the use of exosomes in translational research has certain challenges. They are heterogeneous, and no approach or definite marker exists that can separate exosomes from small microvesicles or exosome sub-populations, which hinders the biological characterization of exosomes as well as treatment standardization. However, the biggest challenge to the clinical translation of exosomes is the technical limitation. The isolation and characterization of exosomes needs to be standardized and simplified to facilitate daily applicability in clinical practice. The potential of exosomes serving as biomarkers for cardiovascular diseases requires large cohort clinical studies, and the vesicles should be thoroughly detected to avoid the misinterpretation of the findings. Another limitation of the current literature on exosome research in cardiovascular diseases is the absence of exosome quantification in control subjects and patients, which is important for the clear interpretation of data. This is because without obtaining the number of exosomes, the data on the exosome components (miRNAs, proteins) would not be normalized to the number of exosomes. In summary, exosome biomarkers are in the early stages of discovery. However, there is a strong possibility that exosomes will become powerful diagnostic markers for determining the progression of cardiovascular diseases in the future.

Exosomes exhibit considerable promise in enhancing diagnostic and therapeutic applications within the realm of cardiovascular medicine, yet numerous issues remain unresolved. With the growing acknowledgment of their utility as cell-free therapeutic agents, the frequency of clinical trials focusing on exosomes has increased. However, the vast majority of these exosome-centric clinical trials are currently ongoing, and the outcomes of these studies not readily accessible in the public domain or through published literature. Consequently, the efficacy and success of clinical interventions employing exosomes remain to be substantiated in forthcoming research endeavors.
